# Examining Motor Anticipation in Handwriting as an Indicator of Motor Dysfunction in Schizophrenia

**DOI:** 10.3389/fpsyg.2022.807935

**Published:** 2022-04-01

**Authors:** Yasmina Crespo Cobo, Sonia Kandel, María Felipa Soriano, Sergio Iglesias-Parro

**Affiliations:** ^1^Department of Methodology of Behavioral Sciences, University of Jaén, Jaén, Spain; ^2^Département Parole et Cognition, Université Grenoble Alpes, Grenoble, France; ^3^Mental Health Unit, St. Agustín Universitary Hospital, Linares, Spain

**Keywords:** handwriting, schizophrenia, motor anticipation, motor alterations, kinematical measurement

## Abstract

Dysfunction in motor skills can be linked to alterations in motor processing, such as the anticipation of forthcoming graphomotor sequences. We expected that the difficulties in motor processing in schizophrenia would be reflected in a decrease of motor anticipation. In handwriting, motor anticipation concerns the ability to write a letter while processing information on how to produce the following letters. It is essential for fast and smooth handwriting, that is, for the automation of graphomotor gestures. In this study, we examined motor anticipation by comparing the kinematic characteristics of the first *l* in the bigrams *ll* and *ln* written on a digitiser. Previous studies indicated that the downstroke duration of the first *l* is modulated by the anticipation of the local constraints of the following letter. Twenty-four adult individuals with diagnosis of schizophrenia and 24 healthy adults participated in the study. The classic measures of duration (sec), trajectory (cm), and dysfluency (velocity peaks) were used for the kinematic analysis of the upstroke (US) and downstroke (DS). In the control group, the duration of the downstroke of the *l* was longer in *ln* than *ll* (US: *ln* = *ll*; DS: *ln* > *ll*) whereas no differences were found for the group with schizophrenia. Likewise, the control group showed a longer DS trajectory for the *l* of *ln* than *ll* in downstrokes, while the group of patients failed to show this effect. These results suggest that the motor alterations in patients with schizophrenia could also affect their ability for motor anticipation.

## Introduction

Experimental and neuropsychological models consider that word writing results from a series of central and peripheral processes that function according to a hierarchical manner (Van Galen, 1991; Bonin et al., 2001; Miceli and Capasso, 2006; Damian and Stadthagen-Gonzalez, 2009; Kandel et al., 2011, 2017; Ellis, 2014). The central processes refer to linguistic processing, such as gearing up the semantic system, syntactic construction, and orthographic retrieval. The peripheral processes point to the motor-related aspects of letter production, where graphomotor planning for handwriting takes place. They are involved in the selection of allographs, where motor program retrieval takes place, local parameter adjustments and muscular activation leading to the production of letters (Bertram et al., 2015). The present research focuses on the latter, lower level peripheral aspects of writing. Of particular interest for the purpose of the study is the phenomenon of motor anticipation. Motor anticipation refers to the ability to predict future behaviors, related to the perception of trajectories and synchronization of movements (Oña et al., 1999). Motor anticipation can be considered as a relevant motor-perceptual process in most learned behaviors (Kandel et al., 2000), and its dysfunction can be linked to some motor disorders as the ones observed in schizophrenia (Finney, 2015).

Traditionally, research has employed diverse measures of handwriting in the study of motor symptoms in psychotic disorders (Caligiuri et al., 2015; Gawda, 2016). Motor symptoms were first studied in psychosis as side effects of the antipsychotic treatment (Chengappa et al., 1994; Simpson and Lindenmayer, 1997). However, recently, the role of motor symptoms in psychotic disorders has been revised (Jahn et al., 2002; Rogowska et al., 2003) and they have been considered as a core feature in the evaluation and the prognosis of the disorder. In this regard, they have been detected in antipsychotic naïve patients with a first psychotic episode (Peralta et al., 2010), and handwriting measures have revealed spontaneous motor abnormalities even in individuals at high risk of psychosis who have never been in pharmacological treatment (Dean et al., 2013, 2014). The present study examined a novel measure of handwriting, motor anticipation in handwriting, in order to gain insight on motor dysfunctions in schizophrenic patients.

Planning and execution of complex sequences of movements involve a significant amount of look-ahead. In fact, units of motor action being executed often carry the imprint of yet-to-be-executed units. More precisely, motor anticipation in fine motor skills, such as writing, concerns the ability to write a letter while processing information on how to produce the following letters. It is essential for fast and smooth handwriting, that is, for the automation of graphomotor gestures. In this study, schizophrenic patients and healthy adults had to write cursive letters on a digitizer. We compared the productions of the two groups on their ability to anticipate forthcoming motor sequences in cursive handwriting.

**Figure 1:**
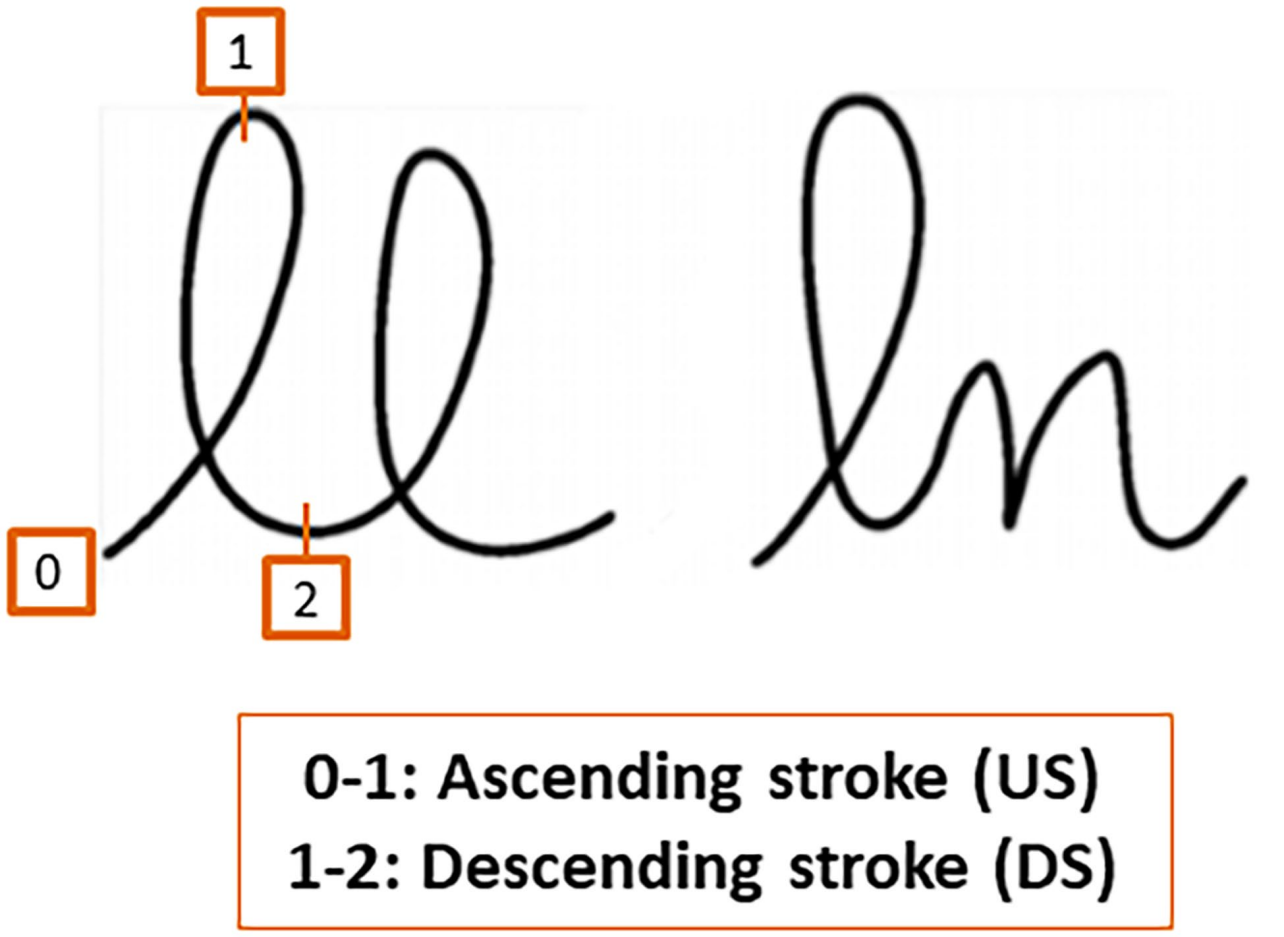
Bigrams *ll* and *ln*. The upstroke refers to the ascending stroke and the downstroke to the descending stroke.

Previous studies carried out by Orliaguet and Boë (1990) with healthy adults indicated that the anticipation of the local production constraints of the following letter modulates the spatio-temporal course of the current movement. These authors compared the kinematic characteristics of the first *l* in bigrams *ll* and *ln* written on a digitizer (Figure 1).

They observed that the anticipatory processing of changes in size and rotation direction of the *n* increased the downstroke duration of the *l* with respect to the first *l* of *ll*, where the same motor program is reproduced (Orliaguet and Boë, 1990; Boë et al., 1991). Furthermore, Kandel and Perret (2015) studied motor anticipation in children at the period of writing automation. Children of ages 8, 9, and 10 years wrote bigrams *ll, le, ln* in cursive writing on a digitizer. They analyzed the duration, trajectory, and dysfluency of the first letter *l*, both on the upstroke and downstroke. They found that at all ages, the *l*’s downstroke duration was shorter for *ll* than *le* and the latter was in turn shorter than *ln*. This modulation of the *l* duration reflects that during this downstroke movement, the children were processing in advance the following letter. The measures of the length of the paths of the children’s productions further revealed that the trajectories of the *l* of *ll* were shorter than those of the l of bigrams *le* and *ln*. The dysfluency data—measured as the number of absolute velocity peaks in the velocity profile for each stroke—indicated that at age 8, dysfluency values were equivalent for upstrokes and downstrokes, whereas children of ages 9 and 10 years old showed more dysfluency on downstrokes than upstrokes. This experiment suggested that learning to anticipate in handwriting production requires: (a) rendering the movements to produce the upstroke constant; and (b) modulating the downstroke as a function of the spatial characteristics of the following letter. The pattern of movement time data suggested that motor anticipation would start to be adult-like at around age of 9. In other words, motor anticipation is already present at age 8 and is a core component of the automation process of handwriting production.

Motor anticipation has also been studied in Parkinson’s patients (Bidet-Ildei et al., 2011). In this study, motor anticipation of the control group was compared with the clinical sample before and after a treatment phase of dopaminergic medication or bilateral deep brain stimulation. The results showed that the control group participants exhibited signs of anticipation whereas Parkinson’s patients did not. More specifically, the downstroke duration of the first *l* of the healthy adults increased as the constraints of the following letter increased, such that (*ll* < *le* < *ln*). In the group of patients, there were no differences in the duration of the downstroke between different conditions. However, after treatment, the patients did exhibit a decrease of the *l* downstroke duration when it was followed by another *l*.

As mentioned above, motor anticipation is a key process for handwriting automation. The production of a movement while anticipating the requirements of the following motor sequence facilitates a smooth handwriting and, as motor demands are reduced, more cognitive resources can be devoted to higher order processes, such as linguistic or conceptual processes. Therefore, a poor motor anticipation would result in poor execution of handwriting and other cognitive–motor tasks (Lozano and Acosta, 2009; Blanchard et al., 2011; Fett et al., 2011). This can be observed in some mental disorders, such as in schizophrenia, which is characterized by a wide variety of cognitive and motor deficits. Besides this, schizophrenia is associated with increased involuntary movements (Pappa and Dazzan, 2009) and with neurological abnormalities in sensory integration, motor coordination, sequencing complex motor acts, and primitive reflexes (Bombin et al., 2005). Therefore, motor-related deficits associated with schizophrenia could affect the processes involved in automation during handwriting. The main objective of the present research was to examine motor anticipation in handwriting in schizophrenia. We hypothesized that patients with schizophrenia would not be able to anticipate the production of forthcoming movements. This would result in an absence of modulation of the kinematic patterns of the downstroke of the first letter of the sequence.

As in previous experimental studies on motor anticipation, we followed the methodology presented by Orliaguet and Boë (1990). We measured motor anticipation by comparing the kinematic characteristics of the first *l* in the bigrams *ll* and *ln*. The classic measures of duration (movement time in seconds), trajectory (path in cm), and dysfluency (number of velocity peaks) were used for the kinematic analysis of the upstroke (US) and the downstroke (DS; see Figure 1). Motor anticipation would be reflected in a modulation of the kinematic parameters of the DS of the first letter, depending on the following letter (*l* vs. *n*). As in the studies with adults (Orliaguet and Boë, 1990; Bidet-Ildei et al., 2011) and children (Kandel and Perret, 2015), the bigram was written in cursive handwriting on a digitizing tablet. We predicted that the difficulties in the cognitive processes involved in fine motor production in schizophrenia would be reflected in a decrease or absence of this modulation.

## Materials and Methods

### Participants

Twenty-four adult individuals attending the Mental Health Day Unit at the University St. Agustin Hospital (Spain) participated in the study. Inclusion criteria were ICD-10 diagnosis of schizophrenia (F20), and age between 20 and 55 years old (*M* = 37.29; SD = 9.58). Diagnosis of participants was made using a semi-structured interview (SCID-I) according to ICD-10 criteria by the psychiatrist or clinical psychologist in charge of the patient. Out of the 24 participants, 17 (70.8%) were male. Twenty-two participants were right-handed whereas 2 were left-handed. Their mean illness duration was 15.36 years (SD = 10.11). Due to the fact that in Spain there have been different education regulations in the last years, we transformed the academic degree reported by the participants in the number of years needed to obtain it. According to this criterion, the average number of years in the formal education system in this group was 10.79 (SD = 4.48 years). To try to better characterize the educational level of the participants, we categorized the number of years in the educational system into three other categories: low educational level (from 0 to 6 years in the formal educational system), medium educational level (from 7 to 12 years), and high educational level (more than 13 years). According to these categories, the sample of patients included 12.5% with a low educational level, 62.5% with a medium level, and 25% with a high level. There were no patients who suffered from Tardive Dyskinesia: All patients had absent or minimal symptomatology (a score of 0 or 1 in the items of the AIMS). In order to compare doses of antipsychotic treatment, we used Chlorpromazine equivalence (CPZE). CPZE is defined as the dose of a drug which is equivalent to 100 mg of oral dose of chlorpromazine (779.37, SD = 419.28).

The SAS rating scale was used for assessment of drug-induced parkinsonism (Simpson and Angus, 1970). This scale is used in both clinical practice and research settings, and it is composed of 10 items: one item measures gait (hypokinesia), six items measure rigidity, and three items measure glabella tap, tremor, and salivation. For each item, the severity of the symptoms was rated from 0 (none) to 4 (severe). A score of 1 in an item indicated the presence of motor symptoms in a mild form. A mean global score of 3 or more in the full test was used as a threshold to indicate the presence of the extrapyramidal symptoms in a mild form (Ayehu et al., 2014). The mean obtained in our sample was 3.21 (SD = 5.09).

To assess clinical symptoms of schizophrenia, we applied the Spanish version (Peralta and Cuesta, 1994) of the Positive and Negative Syndrome Scale (PANSS; Andreasen and Olsen, 1982; Kay et al., 1987). The PANSS is a 30-item rating instrument comprising three subscales: the seven-item Positive Symptoms subscale (PANSS-P), the seven-item Negative Symptoms subscale (PANSS-N), and the 16-item General Psychopathology subscale (PANSS-G). All 30 items are rated on a seven-point scale (1 = absent to 7 = extreme). Obtained results were *M* = 16.04, SD = 5.39 for PANSS-P, *M* = 20.22, SD = 6.96 for PANSS-N, and *M* = 33.86, SD = 10.00 for PANSS-G.

In order to exclude patients with gross motor dysfunctions, we measured finger and hand dexterity with the Purdue Pegboard test (Tiffin and Asher, 1948; Tiffin, 1968). This board consists of two parallel rows of 25 holes each. Pins (pegs) are located at the extreme right-hand and left-hand cups at the top of the board. Metal collars and washers occupy the two middle cups. In the first three subtests, the subject places as many pins as possible in the holes, first with the preferred hand (dominant), then with the non-preferred hand (non-dominant), and finally with both hands, within a 30-s time period. To test the right hand, the subject must insert as many pins as possible in the holes, starting at the top of the right-hand row (*M* = 13.74, SD = 8.65). The left-hand test uses the left row (*M* = 10.73, SD = 2.95). Both hands then are used together to fill both rows top to bottom (*M* = 8.01, SD = 2.25). In the fourth subtest, the subject uses both hands alternately to construct “assemblies,” which consist of a pin, a washer, a collar, and another washer. The subject must complete as many assemblies as possible within 1 min (*M* = 28.59, SD = 9.23). We did not exclude any participants based on their scores on this test.

For the control group, 24 adults were recruited from the University of Jaén and an adult school of Jaén. The inclusion criterion was that age was between 20 and 60 years (*M* = 36.83 years old; SD = 12.83 years old). Out of the 24 participants, 14 were male. All of the participants were right-handed. Regarding educational level, 3 participants had low level, 8 medium and 19 participants had high education level (*M* = 13.25, SD = 10). Importantly, there were no significant differences between groups on age (*t* = 1.73, *p* = 0.08), sex (*χ*^2^ = 0.82, *p* = 0.36) or educational level considered either as the number of years in the educational system (*t* = 1.1.59; *p* = 0.12) or categorized in low, medium or high level (*χ*^2^ = 4.70, *p* = 0.09).

Exclusion criteria for both groups were: concurrent diagnosis of neurological disorder, concurrent diagnosis of substance abuse, history of developmental disability, inability to sign informed consent or vision disorders (those vision disorders which, although corrected by glasses or contact lenses, suppose a loss of visual acuity, e.g., cataracts). In addition, an exclusion criterion for the control group was the diagnosis of a mental disorder (according to verbal reports from participants).

All participants gave their written informed consent according to the Declaration of Helsinki and the Ethics Committee on Human Research of the Hospital approved the study.

### Procedure and Data Analysis

Participants were asked to perform an easy and brief handwriting task. A A4 paper was affixed to the surface of a WACOM (Intuos pro small) digitizing tablet with dimensions of 269 × 170 × 8 mm (10.6 × 6.7 × 0.3 in), with an active area of 160 × 100 mm (6.3 × 3.9 in) and a resolution of 5,080 lpi. The different bigrams (*ll, ln*) were presented randomly on a computer screen, and participants were required to write the bigrams using this paper, with a Wacom Pro Pen 2 (KP504E) digital pen with 8192 levels of pressure sensitivity. Handwriting tasks were carried out individually. The task had no time limit.

We measured three dependent variables: the time per stroke (Duration, seg.), the path of the pen for each stroke (Trajectory, cm), and the number of velocity peaks (Disfluency).

For each of these dependent variables, we run separate mixed models with Group, Direction and Bigram as independent variables. Random intercepts were included for subjects. Analyses were done in R [R Core Team (2020)] using the lmer() function of the lme4 package (Bates et al., 2015). We utilized the restricted maximum likelihood as the estimation procedure and the Welch–Satterthwaite (Luke, 2017) approximation of the degrees of freedom because of our relatively small sample size (Gumedze and Dunne, 2011).

## Results

### Duration

**Figure 2:**
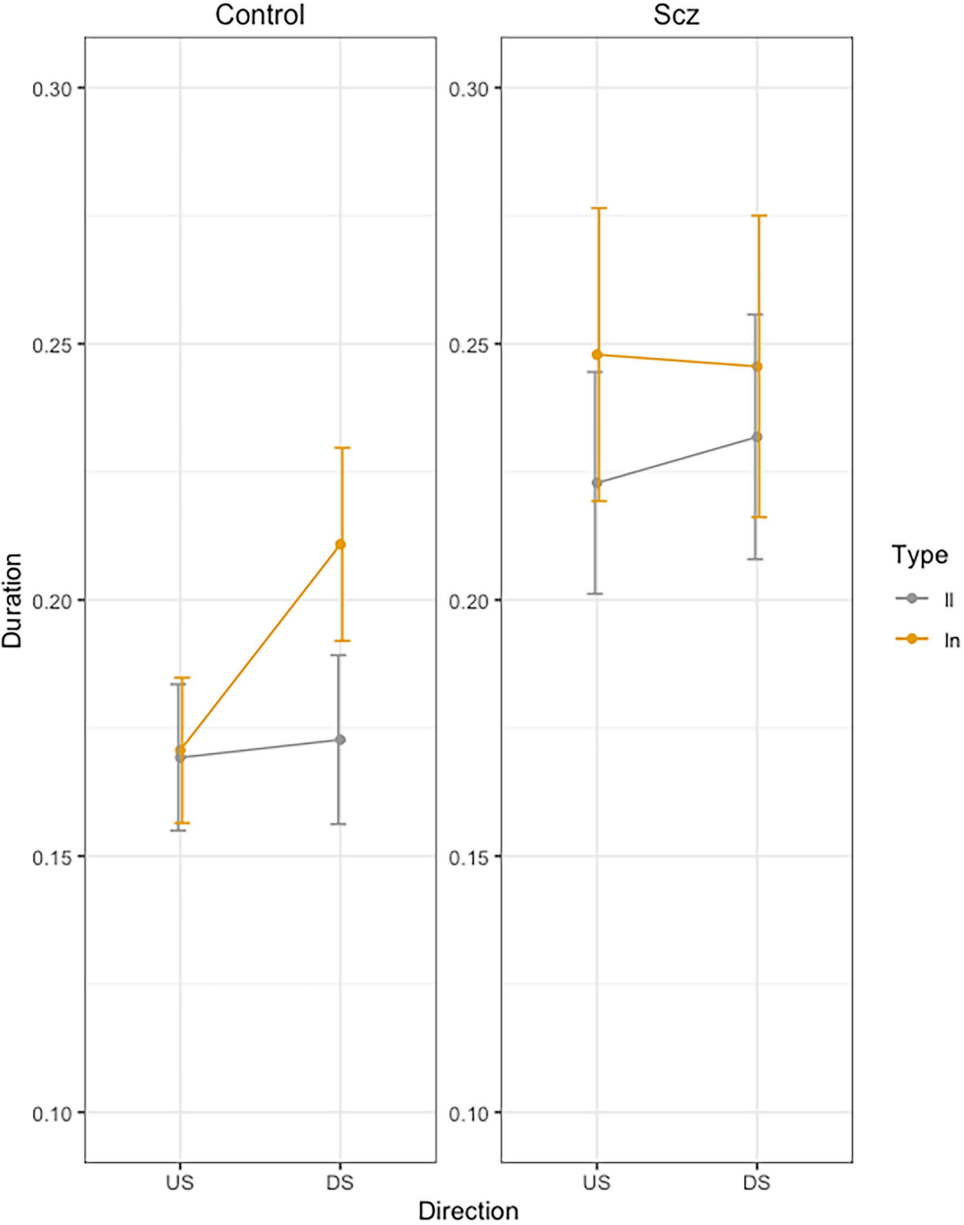
Mean movement time per stroke (Duration, sec.) across trials, as a function of group (Schizophrenia-SCZ vs. Control-CTRL), stroke direction (downstroke-DS vs. upstroke-US), and type of bigram (*ll* vs. *ln*). Error bars represent standard error.

Figure 2 presents mean movement time per stroke across trials, as a function of group (Schizophrenia-SCZ vs. Control-CTRL), stroke direction (downstroke-DS vs. upstroke-US), and type of bigram (*ll* vs. *ln*). The ANOVA on duration revealed a significant effect was found for Direction [*F*(1, 138) = 4.83, *p* = 0.029, $\eta_{p}^{2}=0$ .033], indicating a longer duration for DS (*M* = 0.21, *SD* = 0.01) than US (*M* = 0.20, *SD* = 0.01). A significant effect was also found for Bigram [*F*(1, 138) = 11.69, *p* < 0.01, $\eta_{p}^{2}=$ 0.078], indicating a longer duration for *ln* (*M* = 0.21, *SD* = 0.11) than for *ll* (*M* = 0.19, *SD* = 0.09). The interaction Group by Direction by Bigram was also significant [*F*(1, 138) = 4.39, *p* = 0.037, $\eta_{p}^{2}=$ 0.031]. No other effects were significant.

In order to analyze the Group by Direction by Bigram interaction, we conducted pairwise comparisons using emmeans function in R (Russell Lenth, 2020). In the control group, we found no significant differences in US between LL and LN (*t <* 1), but we found significant longer duration for LN than for LL (*t* = −3.32, *p* = 0.24) in DS. In the schizophrenia group, we found no significant differences were found between LL and LN (*t <* 1) neither in US (*t* = −2.18, *p* = 0.36) nor in DS (*t* = −1.20, *p* = 0.93).

### Trajectory

**Figure 3:**
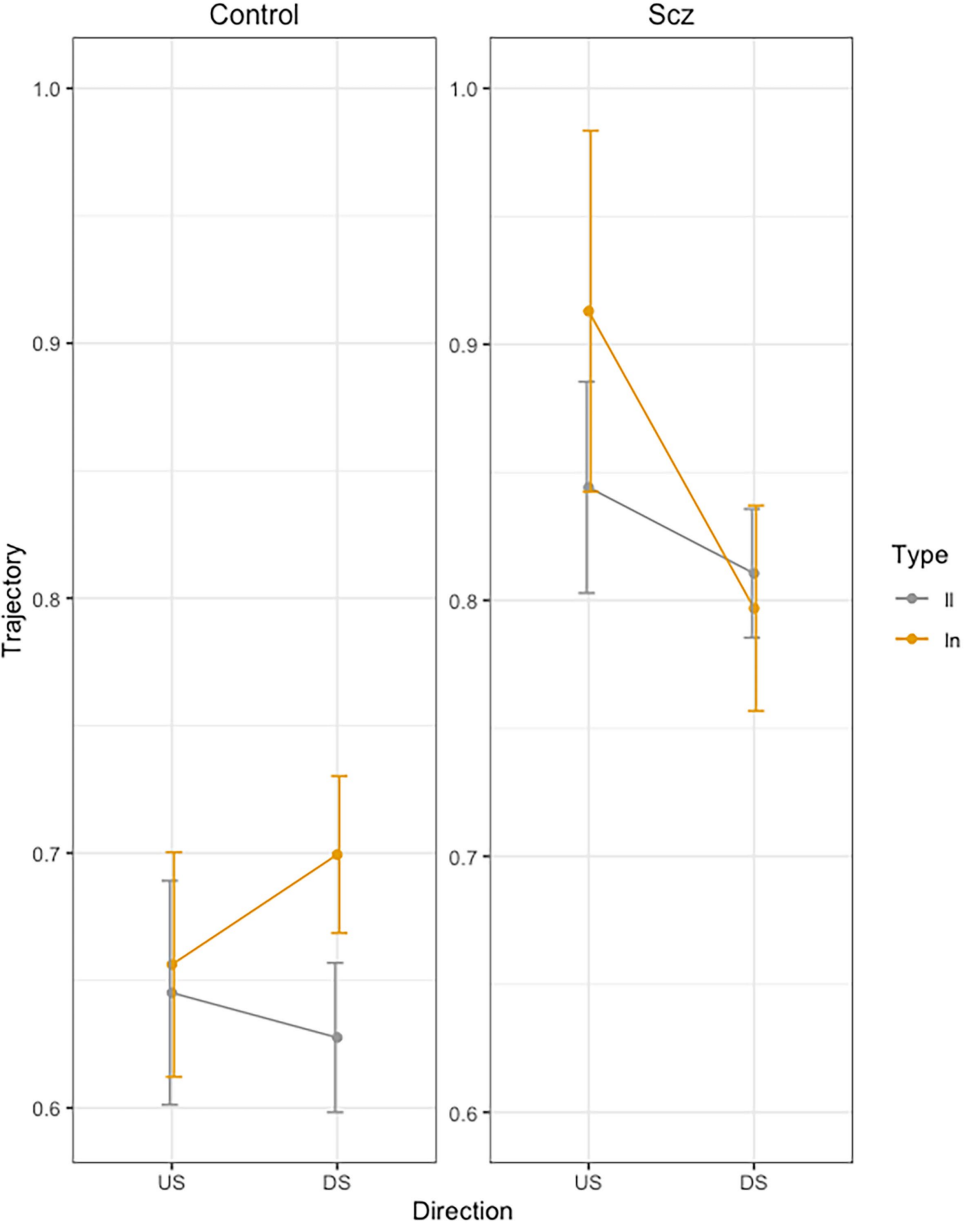
Mean trajectory (path in cm of the pen) for each stroke across trials, as a function of group (Schizophrenia-SCZ vs. Control-CTRL), stroke direction (downstroke-DS vs. upstroke-US), and type of bigram (*ll* vs. *ln*). Error bars represent standard error.

Figure 3 presents the mean Trajectory each stroke across trials, as a function of group (Schizophrenia-SCZ vs. Control-CTRL), stroke direction (downstroke-DS vs. upstroke-US), and type of bigram (*ll* vs. *ln*). Error bars represent standard error. The ANOVA showed a significant effect of Group [*F*(1, 46) = 12.68, *p* < 0.01, $\eta_{p}^{2}=$ 0.216] indicating longer trajectory in the SCZ group (*M* = 0.84, SD = 0.23) than the Control group (*M* = 0.65, SD = 0.18). A marginally effect was also found for Type of bigram [*F*(1, 138) = 3.64, *p* = 0.058], indicating a longer trajectory for *ln* (*M* = 0.76, SD = 0.25) than for *ll* (*M* = 0.73, SD = 0.19). The interaction Group by Direction was significant [*F*(1, 138) = 5.87, *p* < 0.016, $\eta_{p}^{2}=.040$ ]. The interaction Group by Direction by Bigram was also significant [*F*(1, 138) = 3.91, *p* < 0.049, $\eta_{p}^{2}=.027$ ]. No other effects were significant.

In order to analyze the Group by Direction by Bigram interaction, we conducted pairwise comparisons using emmeans function in R (Russell Lenth, 2020). In the control group, we found no significant differences in DS between LL and LN (*t* = −1.98, *p* = 0.49), but we found significant longer duration for LN than for LL (*t* = −3.32, *p* = 0.24) in US. In the schizophrenia group, we found no significant differences were found between LL and LN (*t <* 1) neither in US (*t* = −2.18, *p* = 0.36) nor in DS (*t* = −1.20, *p* = 0.93).

### Disfluency

**Figure 4:**
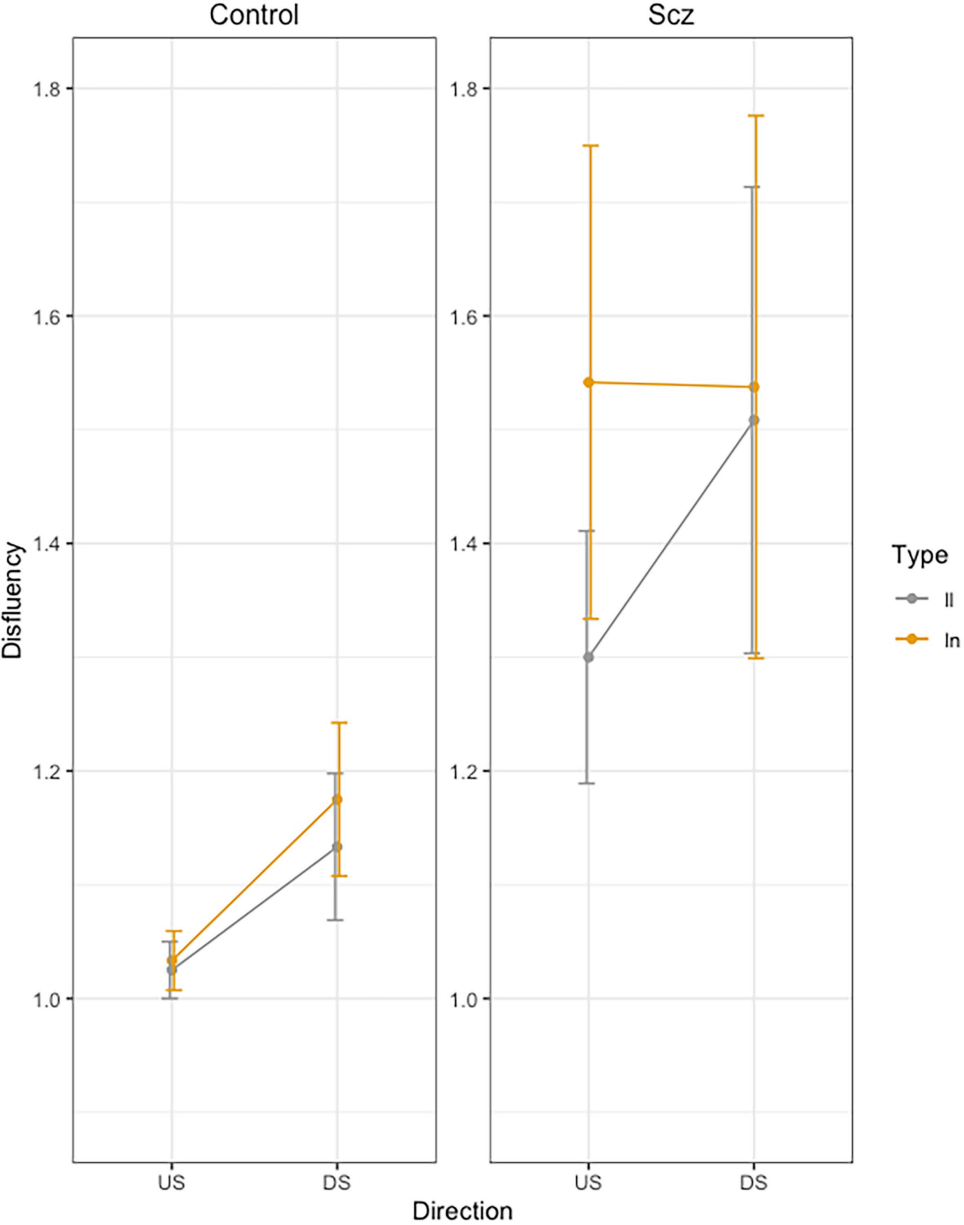
Mean Dysfluency (number of velocity peaks) across trials, as a function of group (Schizophrenia-SCZ vs. Control-CTRL), stroke direction (downstroke-DS vs. upstroke-US), and type of bigram (*ll* vs. *ln*). Error bars represent standard error.

Figure 4 presents mean Dysfluency values as a function of group (Schizophrenia-SCZ vs. Control-CTRL), stroke direction (downstroke-DS vs. upstroke-US), and type of bigram (*ll* vs. *ln*). The results yielded a significant Group effect [*F*(1, 48) = 4.34, *p* = 0.042, $\eta_{p}^{2}=.082$ ] indicating more dysfluency in the SCZ group (*M* = 1.47, *SD* = 0.98) than in the Control group (*M* = 1.09, *SD* = 0.24). A significant effect was also found for Direction [*F*(1, 144) = 6.31, *p* = 0.013, $\eta_{p}^{2}=.041$ ], indicating more velocity peaks for the DS (*M* = 1.33, SD = 0.81) than US (*M* = 1.22, SD = 0.61). No other effects were significant.

**Figure 5:**
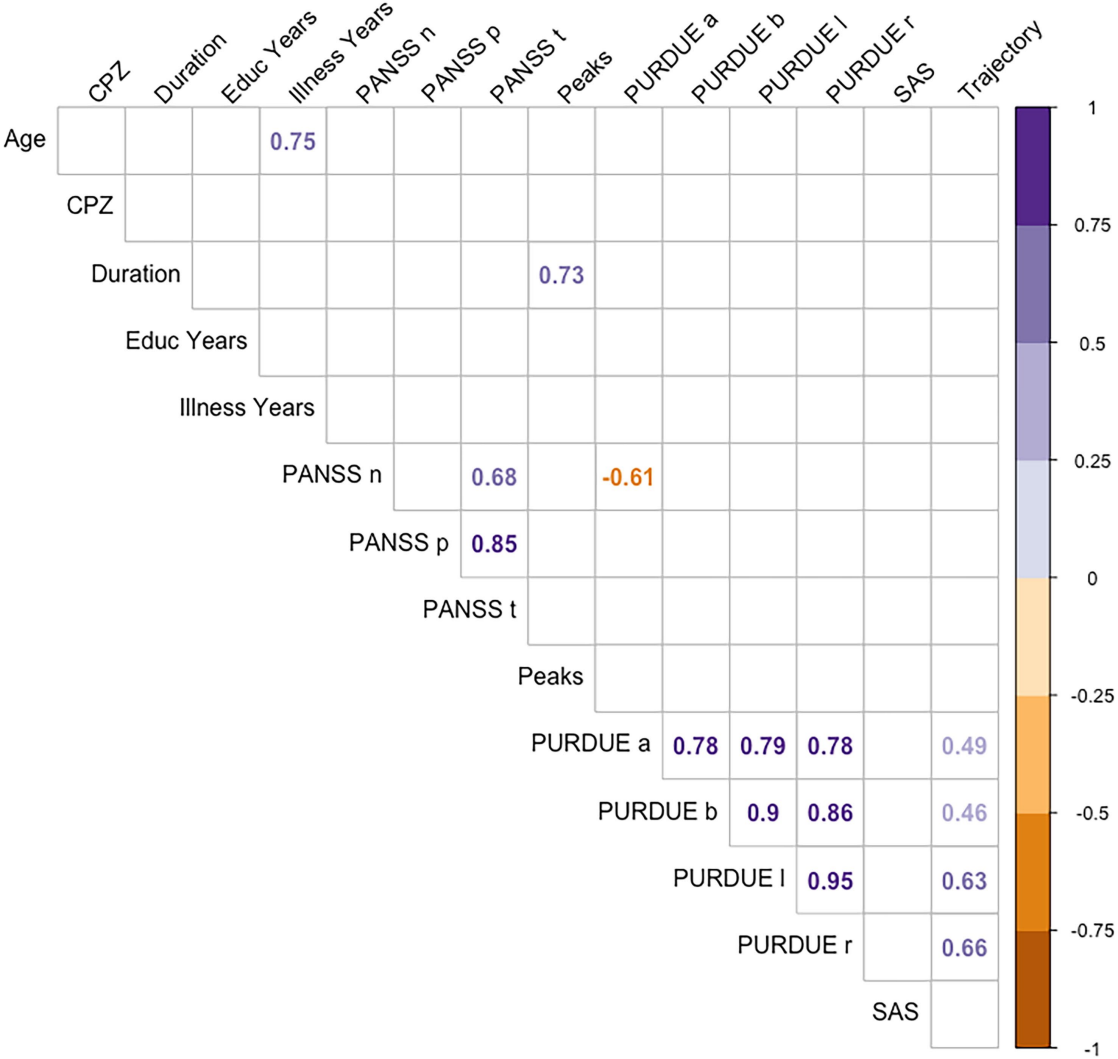
Spearman coefficients for CPZ, educational level, illness years, PURDUE, SAS, PANSS, and the handwriting variables.

Finally, correlations were carried out between the different measures from motor evaluation scales, psychopathology scales, and other characteristic variables of the disorder (illness duration, educational level or pharmacological treatment doses), and the kinematic measures of handwriting (see Figure 5). We found no significant relationships between kinematic measures of handwriting and the rest of variables, except for Trajectory, which correlated with motor functioning values from the Purdue test: patients with a worse motor function showed longer trajectories in handwriting.

## Discussion

Handwriting becomes automatic with practice. Motor automation is essential to free up cognitive and attentional resources for the rest of the components of handwriting: conceptual planning, syntactical construction, lexical selection, etc. In adult cursive handwriting, movements are smooth and continuous. Part of this continuity is due to motor anticipation. It allows for the programming of the graphomotor constraints of the following stroke while executing the previous one. Since letters vary in size and direction of the stroke when they are written in lowercase, the anticipation of these variations requires a supplementary cognitive load while preparing the production of the following letter. This anticipation modulates the spatio-temporal course of the production movement, which can be observed in some kinematic parameters of handwriting. Based on this idea, and following the experimental paradigm presented by Orliaguet and Boë (1990), the present research evaluated motor anticipation in patients with schizophrenia and controls. We measured the kinematic variations in the upstroke (US) and downstroke (DS) of the first letter *l* of a bigram, as a function of the graphomotor constraints of the following letter. Motor anticipation implies a modulation of the duration of the descending stroke as a function of size and rotation direction of the following letter (e.g., Kandel and Perret, 2015). If the forthcoming letter requires a change in size and direction from one letter to the other as in *ln*, the cognitive load is usually reflected in an increase in the duration of the descending stroke compared to a bigram in which the same motor program is repeated as in *ll*.

The results supported our main hypothesis: patients with schizophrenia did not exhibit any sign of motor anticipation. In the control group instead, changes in letter size and direction increased the downstroke duration of the first *l*, whereas for the upstrokes it remained unaffected (*l* US: *ln* = *ll*; *l* DS: *ln* > *ll*). The fact that the duration of the downstroke increases as the spatial parameters of the following letter change, is interpreted as a sign of motor anticipation. These results contrast with those of the schizophrenia group: no differences were found in duration according to the type of bigram (*ll* = *ln*) or stroke direction (US = DS); interaction type of bigram and direction failed to reach significance (US: *ln* = *ll*; DS: *ln* = *ll*).

Stroke duration seems to be the most sensitive kinematic measure of motor anticipation according to previous research (Orliaguet and Boë, 1990; Bidet-Ildei et al., 2011; Kandel and Perret, 2015). There are several brain structures involved in handwriting that are disrupted in schizophrenia, but it is the basal ganglia that have been mostly related to the deautomatization of writing. This dysfunction of the basal ganglia can result in a general impairment in motor planning and coordination. This impairment, associated to a delay in corrective movements, could cause a segmentation of sequential movements and disrupt motor anticipation in handwriting (Pantelis et al., 1992; Lange et al., 2006; Smiley-Oyen et al., 2007; Bidet-Ildei et al., 2011).

The trajectory results also reflect this kind of impairment in motor anticipation in the patients. The control group produced longer trajectories for the DS of the *l* of *ln* than *ll* and no differences in US, while the group of patients failed to show this effect. These results can be interpreted as another sign of motor anticipation in healthy people compared to patients with schizophrenia. Although previous studies have not found this trajectory modulation, our data clearly support that anticipation can also be reflected in the trajectory of the stroke. We also found that the strokes in the schizophrenia group were in general longer than the strokes in the control group. This result is consistent with previous studies that report the presence of macrography in schizophrenia (Gallucci et al., 1997; Caligiuri et al., 2015; Kömür et al., 2015). Of particular interest are those studies that relate a decrease in the size of handwriting with dopamine D2 receptor occupancy after risperidone treatment (Kuenstler et al., 1999; Regenthal et al., 2005). Future research could deepen these results.

Finally, the fluency measure (i.e., the number of velocity peaks) was not sensitive to motor anticipation, although the patients exhibited more dysfluency than controls. This is in line with previous studies revealing more dysfluent movements in handwriting in patients with schizophrenia and related disorders (Lohr and Caligiuri, 2006; Caligiuri et al., 2015; Crespo et al., 2019). In general, dysfluent movements occur when the muscles that coordinate the movements receive dysregulated signals from the basal ganglia (Caligiuri et al., 2009, 2010). Whether operationalized as absolute velocity peaks or acceleration changes over time, dysfluency in handwriting is always present in schizophrenia. The fact that in our study the schizophrenia group presented greater trajectory length and more dysfluency is a sign of a general motor impairment in this disorder.

It is also noteworthy that we did not observe any relation between psychotic symptoms, pharmacological treatment, and demographic variables and kinematic measurements. We can therefore discard that the groups differences in motor anticipation could be due to psychomotor slowing related to the evolution of the disorder, It is also noteworthy that we did not observe any relation between psychotic symptoms, pharmacological treatment, and demographic variables and kinematic measurements. We can therefore discard that the groups differences in motor anticipation could be due to psychomotor slowing related to the evolution of the disorder, but not to the pharmacological treatment or another demographic variables. However, we think that the absence of motor anticipation in patients is not caused by pharmacological treatment. First, drug induced parkinsonism consists of a number of motor symptoms, such as rigidity, bradykinesia, and tremor, but, to our knowledge, deficits in motor anticipation have never been considered as a symptoms of parkinsonism. Parkinsonism could be reflected in some characteristics of patients handwriting, for example, velocity, trajectory or fluency, but it would be reflected in a group effect, not in an interaction between these characteristics and bigram or direction. That is, parkinsonism could be reflected in patients handwriting being slower than controls handwriting, but, in our opinion, it has no sense that a difference between patients and controls only in upstrokes but not downstrokes would reflect parkinsonism. Thus, we interpreted that, in our study, results in fluency reflect parkinsonism (idiopathic or drug-induced), but results in duration and trajectory (where we found and interaction between group, direction, and bigram) reflect a specific deficit in motor anticipation.

In summary, patients with schizophrenia fail to show the typical motor anticipation effect in handwriting, evidenced by a modulation of duration of the first letter of the bigram as a function of spatial constraints of the second letter. In a broader sense, this research constitutes further evidence in favor of the analysis of handwriting as a quantitative, objective, and reliable tool to detect motor alterations in schizophrenia. Traditionally, the assessment of motor alterations has been carried out by means of observation scales, such as the Simpson-Angus Scale (SAS) and the Abnormal Involuntary Movements Scale (AIMS). However, some studies have highlighted the insufficient predictive value of these scales, a low specificity (Blanchet et al., 2012) and an acceptable reliability only if the evaluation is performed by trained evaluators (Lane et al., 1985; Tonelli et al., 2003). The analysis of handwriting on digitizing tablets allows us to extract a number of handwriting measures that can reveal different cognitive and motor processes disrupted by the disorder.

## Data Availability Statement

The datasets analysed during the current study are available from the corresponding author on reasonable request.

## Ethics Statement

The studies involving human participants were reviewed and approved by Comité de ética de la investigación de Jaén, Junta de Andalucía. The patients/participants provided their written informed consent to participate in this study.

## Author Contributions

All authors listed have made a substantial, direct and intellectual contribution to the work, and approved it for publication.

## Funding

This research was funded by Junta de Andalucía (Biomedical and Heath Science research project PI-0410-2014, PI-0386-2016 and AP-0033-2020-C1-F2) and PID2019-105145RB-I00/Agencia Estatal de Investigación AEI/10.13039/501100011033. The funders had no role in study design, data collection, and analysis, decision to publish, or preparation of the manuscript.

## Conflict of Interest

The authors declare that the research was conducted in the absence of any commercial or financial relationships that could be construed as a potential conflict of interest.

## Publisher’s Note

All claims expressed in this article are solely those of the authors and do not necessarily represent those of their affiliated organizations, or those of the publisher, the editors and the reviewers. Any product that may be evaluated in this article, or claim that may be made by its manufacturer, is not guaranteed or endorsed by the publisher.
